# Habitat complexity reduces parasitoid foraging efficiency, but does not prevent orientation towards learned host plant odours

**DOI:** 10.1007/s00442-015-3346-y

**Published:** 2015-05-23

**Authors:** H. M. Kruidhof, A. L. Roberts, P. Magdaraog, D. Muñoz, R. Gols, L. E. M. Vet, T. S. Hoffmeister, J. A. Harvey

**Affiliations:** Population and Evolutionary Ecology Group, University of Bremen, Bremen, Germany; Department of Terrestrial Ecology, Netherlands Institute of Ecology (NIOO-KNAW), Wageningen, The Netherlands; Laboratory of Applied Entomology, Nagoya University, Nagoya, Japan; Laboratory of Entomology, Wageningen University, Wageningen, The Netherlands; Wageningen UR Greenhouse Horticulture, Bleiswijk, The Netherlands

**Keywords:** *Cotesia glomerata*, *Pieris brassicae*, Learning, Semi-field study, Host location

## Abstract

It is well known that many parasitic wasps use herbivore-induced plant odours (HIPVs) to locate their inconspicuous host insects, and are often able to distinguish between slight differences in plant odour composition. However, few studies have examined parasitoid foraging behaviour under (semi-)field conditions. In nature, food plants of parasitoid hosts are often embedded in non-host-plant assemblages that confer both structural and chemical complexity. By releasing both naïve and experienced *Cotesia glomerata* females in outdoor tents, we studied how natural vegetation surrounding *Pieris brassicae*-infested *Sinapis arvensis* and *Barbarea vulgaris* plants influences their foraging efficiency as well as their ability to specifically orient towards the HIPVs of the host plant species on which they previously had a positive oviposition experience. Natural background vegetation reduced the host-encounter rate of naïve *C. glomerata* females by 47 %. While associative learning of host plant HIPVs 1 day prior to foraging caused a 28 % increase in the overall foraging efficiency of *C. glomerata*, it did not reduce the negative influence of natural background vegetation. At the same time, however, females foraging in natural vegetation attacked more host patches on host-plant species on which they previously had a positive oviposition experience. We conclude that, even though the presence of natural vegetation reduces the foraging efficiency of *C. glomerata*, it does not prevent experienced female wasps from specifically orienting towards the host-plant species from which they had learned the HIPVs.

## Introduction

How consumers optimize the location and exploitation of resources in their natural habitats has long underpinned ecological and evolutionary theory (Charnov 1976; Macarthur and Pianka 1966; Vinson 1976). Given that habitats exhibit physical and chemical properties that vary in their complexity, small organisms such as insects may be severely challenged when their resources are scarce in space and time or are embedded in plant patches of nonfocal species. Parasitoid wasps make excellent model organisms to study questions related to resource exploitation. Parasitoids are organisms that develop in or on the bodies of other insects (“hosts”), whereas the adults are free-living. Unlike predators, parasitoids are dependent for their development on a single host individual (Godfray 1994).

To locate suitable hosts after emerging from the cocoon, parasitoids follow a step-wise hierarchical process involving habitat location, host plant location, and host location (Vinson 1976). By using specific information from the hosts’ food plant, as well as from its habitat (i.e. the vegetation that surrounds the hosts’ food plant), parasitoids can circumvent the problem of host scarcity and a low detectability of host-derived cues. Upon damage by herbivores, plants increase their volatile production and change their odour blend composition, resulting in the emission of so-called herbivore-induced plant volatiles (HIPVs) (McCormick et al. 2012; Vet and Dicke 1992). Although less reliable than host-derived cues, HIPVs are more easily detectable (Vet et al. 1991) and can attract parasitoids from larger distances (Braasch and Kaplan 2012; Geervliet et al. 1998a). Parasitoids innately respond to the HIPVs of their hosts’ food plants, and may prefer blends of certain host-infested plant species (Bukovinszky et al. 2005; Geervliet et al. 1996; Gols et al. 2009, 2011) or plant genotypes (Hoballah et al. 2002; Poelman et al. 2009) over others. Innate preferences are expected to be adapted to the most suitable or accessible plant–host complexes over evolutionary time (Vet et al. 1995). For most parasitoid species, however, preferences for plant odours are not fixed throughout their lifetimes, but can change according to experience (Hoedjes et al. 2011; Vet et al. 1990, 1995). Innately less attractive plant odours can be reinforced after one or more positive oviposition experiences into host insects, resulting in a shift in preference towards the learned odour (Vet et al. 1990).

Many parasitoids, especially so-called koinobionts (those that permit continued host development following parasitization (Askew and Shaw 1986)), are highly specialized and will attack only one or a few host species in nature (Althoff 2003; Jancek et al. 2013). The food plants of these hosts are often embedded into plant assemblages that create both structural and chemical complexity. This habitat complexity has, however, been largely ignored when studying parasitoid foraging behaviour. By far the most studies examining tritrophic interactions involving plants, insect herbivores and their parasitoids have been performed under artificial conditions in the laboratory (Hunter 2002; Wäschke et al. 2013). While several field studies have demonstrated that HIPV emission by host plants results in the attraction of natural enemies (De Moraes et al. 1998; Halitschke et al. 2008; Poelman et al. 2009; Thaler 1999), these studies were performed in simple monocultures without the presence of non-host-plant vegetation surrounding the host-infested plants. Yet, some studies indicate that the plethora of volatiles emitted by the non-host plants that form the vegetation can interfere with the olfactory orientation of parasitoids (Randlkofer et al. 2010; Wäschke et al. 2013, but see 2014), resulting in chemical masking of host-plant HIPVs. Furthermore, structural complexity may physically impede parasitoid movement and/or conceal host-infested plants (Casas and Djemai 2002; Gols et al. 2005; Obermaier et al. 2008; Randlkofer et al. 2010).

Several studies have examined the relationship between vegetation diversity and parasitoid abundance, with variable outcomes. While some parasitoid species showed greater presence in habitats with diverse vegetation (Fraser et al. 2007; Vanbergen et al. 2006), other parasitoid species were more abundant in single-species habitats (Bezemer et al. 2010; Langer 1996) or showed no response to habitat species diversity (Waschke et al. Wäschke et al. 2014). In none of these studies, however, were the precise mechanisms underlying the observed abundance patterns disentangled. So far, only a few studies have directly examined the effect of habitat complexity on parasitoid foraging behaviour. Using crop plants, it was found that females of the parasitoid species *Diadegma semiclausum* and *Cotesia glomerata* were less efficient at locating hosts in mixed cultures compared with monocultures (Gols et al. 2005; Perfecto and Vet 2003). Interestingly, this effect disappeared in both study systems after the females gained experience with ovipositing into hosts on the respective host plants (Bukovinszky et al. 2007; Perfecto and Vet 2003). It should, however, be noted that females of some other parasitoid species showed higher foraging efficiencies in mixed cultures than in monocultures (Coll and Bottrell 1996; Perfecto and Vet 2003).

Habitat structural and chemical complexity may not only affect parasitoid foraging efficiency (i.e. the rate at which hosts are encountered) but also the degree to which parasitoids can orient better towards the HIPVs of host plant species they previously had a positive oviposition experience on. Thus far, few studies have considered the effects of non-host plants on parasitoid orientation towards (learned) HIPVs. Here, we used a naturally occurring tritrophic model system to study how natural vegetation surrounding host-infested plants influences (1) the foraging efficiencies of both naïve and experienced females of the parasitoid species *Cotesia glomerata* (Hymenoptera: Braconidae) and (2) their ability to orient specifically towards the HIPVs of host plant species on which they previously had a positive oviposition experience. We chose *C. glomerata* as our model species, as its behaviour and biology have been well studied in the laboratory and in agricultural fields (Geervliet et al. 1998b; Kruidhof et al. 2012; Poelman et al. 2009; Smid et al. 2007; Vos et al. 1998).

## Materials and methods

### Plants

Two plant species, *Sinapis arvensis* L. and *Barbarea vulgaris* L. (Brassicacaea) were selected as model plants. Both species occur naturally in northern Europe and are used as host plants by the large cabbage white *Pieris brassicae* L. (Lepidoptera; Pieridae) (Feltwell 1982). Moreover, they grow in similar habitat types (disturbed areas, roadside verges) and have partially overlapping temporal niches. Seeds from a naturally growing *S. arvensis* population were collected in July 2010 at a road verge near Arnhem, Netherlands, and seeds from *B. vulgaris* were purchased in 2012 from de Cruydt-Hoeck, Nijerberkoop, Netherlands. Seeds were germinated on sterile glass beads and the seedlings were transplanted after 1 week (*S. arvensis*) or 2 weeks (*B. vulgaris*) to 1.1-L pots filled with 450 g of a mixture of potting soil and gravel (80 %: 20 %). Plants were grown in a greenhouse at temperatures of 21 ± 2 °C (day) and 16 ± 2 °C (night), 60 % relative humidity and under a L16:D8 photoperiod. Natural daylight was supplemented by 400-W metal halide bulbs at a distance of 1.5 m. Plants were watered when needed. Three (*S. arvensis*) or four (*B. vulgaris*) weeks after transplanting, when both plant species were still at the vegetative stage, the plants were used in the experiments. At this point, *S. arvensis* plants were on average higher (21 cm) than *B. vulgaris* plants (13 cm), while the average leaf dry weight of *B. vulgaris* (852 mg) was slightly larger than that of *S. arvensis* (701 mg).

### Insects

*Pieris brassicae* is a gregarious specialist herbivore whose larvae exclusively feed on plants producing glucosinolates (Renwick and Lopez 1999). *Cotesia glomerata* L. (Hymenoptera: Braconidae) is a gregarious endoparasitoid that attacks early-instar larvae of pierid butterflies, with *P. brassicae* being its preferred host (Feltwell 1982). *Cotesia glomerata* wasps lay on average 20–30 eggs into a host caterpillar per oviposition event (Gu et al. 2003). Cultures of *P. brassicae* and *C. glomerata* were established from individuals collected from agricultural fields in the vicinity of Wageningen, Netherlands. *Cotesia glomerata* was reared in *P. brassicae* caterpillars, which in turn were reared on Brussels sprout plants (*Brassica oleracea* L. var. *gemmifera* cv. Cyrus, Brassicaceae). Insect rearing was performed in a climate room at 20–22 °C, 50–70 % RH and under a L16:D8 photoperiod. Upon emergence, approximately 50–70 % of the males were removed from the cages to reduce stress on the females but at the same time allow for mating. All wasps were provided with ample water and honey. Only mated, 3- to 5-day-old female *C. glomerata* wasps were used in the experiments. *Pieris brassicae* caterpillars used for the experiments were transferred as eggs to *S. arvensis* or *B. vulgaris* plants.

### Experimental setup

Parasitoid foraging trials were carried out in 12 tents (3 × 3 m and 2 m high, made of 0.6-mm insect screen) at the experimental garden of the Netherlands Institute of Ecology (Wageningen, Netherlands) over the course of 5 weeks in August and September 2012. Two repetitions were carried out simultaneously each week. As only one repetition could be performed during one of the weeks due to a low number of available *C. glomerata* females, the total number of repetitions amounted to 9. Half of the tents were placed on bare soil that was covered with a soil cover cloth to prevent weed growth (no background vegetation), and the other half of the tents were placed on diverse natural vegetation (natural background vegetation ; see Table 1). Each tent contained a total of twelve host plants; three pairs of *P. brassicae*-infested *B. vulgaris* plants and three pairs of *P. brassicae*-infested *S. arvensis* plants that were alternated in a circle 2 m in diameter. Plant pairs were positioned at a distance of 1 m from each other and from the parasitoid release point in the middle of the circle. Potted host plants were transferred from the greenhouse to the tents and placed in holes in the ground so that the upper rim of the pot was level with the soil. The exact position of the host plant species in the tents was shifted between repetitions but was kept the same between treatments within each repetition in order to control for placement biases from environmental factors such as sun or wind.

**Table 1:** Plant species composition and average soil cover (%) in tents with natural background vegetation

Plant species	Average soil cover (%)	% of tents with species
*Ranunculus repens* (creeping buttercup)	31.9	100
*Trifolium pratense* (red clover)	14.8	100
*Poa pratensis* (smooth meadow-grass)	13.3	100
*Dactylis glomerata* (cocksfoot)	12.9	100
*Tripleurospermum maritimum* (scentless mayweed)	9.1	100
*Plantago major subsp. Major* (greater plantain)	7.1	100
*Holcus lanatus* (Yorkshire fog)	4.8	100
*Juncus bufonius* (toad rush)	4.3	50
*Juncus effusus* (soft rush)	3.3	100
*Epilobium parviflorum* (hoary willowherb)	3.2	100
*Medicago lupulina* (black medick)	2.8	100
*Conyza canadensis* (Canadian horseweed)	0.9	100
*Melilotus altissimus* (tall melilot)	0.8	100
*Echinochloa crus*-*galli* (barnyardgrass)	0.7	83
*Plantago lanceolata* (ribwort plantain)	0.6	33
*Rumex obtusifolius* (broad-leaved dock)	0.5	33
*Jacobaea vulgaris* (tansy ragwort)	0.3	17
*Lytrum salicaria* (purple loosestrife)	0.1	17
Total	111.2	

For the foraging trials, *C. glomerata* females belonging to one of three different conditioning treatments were released in groups of 8 wasps in the middle of the tents and allowed to forage for a period of 3 h. Although the parasitoids had been provided with ample honey and water before they were released, two Petri dishes with additional drops of honey and water-containing cotton wool were placed in each tent. At the end of the foraging period, all caterpillars were retrieved and dissected to check for the presence of *C. glomerata* eggs. Per tent the number of each host plant species containing parasitized hosts was recorded per tent, as well as the number of caterpillars that were parasitized within each host patch. As each tent was reused after 1 week, we checked that *C. glomerata* females were still present in the tents with diverse vegetation 6 days after the previous foraging trial. For this purpose, ten *P. brassicae*-infested *B. vulgaris* and ten *P. brassicae*-infested *S. arvensis* plants were randomly distributed among the tents. After a 4-h period, all of the caterpillars were collected and dissected. As none of the caterpillars contained parasitoid eggs, we assumed that the foraging trials were not affected by interference from previously released *C. glomerata* females.

### Conditioning procedure

Three groups of *C. glomerata* females with different types of experience were released in each foraging trial. One group consisted of naïve females that did not receive any experience with host plant odours. The other two groups of females received a differential conditioning procedure (Scherer et al. 2003). For one group, this consisted of one positive conditioning event on a *S. arvensis* plant followed by a negative conditioning event on a *B. vulgaris* plant (see detailed explanation below). The other group of females received the reciprocal differential conditioning procedure, consisting of one positive conditioning event on a *B. vulgaris* plant followed by a negative conditioning event on a *S. arvensis* plant. In this way, females from both conditioning procedures received identical exposure to the odours of both host plant species as well as the reward, with the connection between these stimuli being the sole difference. In the foraging trials, associative learning can thus be inferred from any systematic differences in parasitization rates of host caterpillars on the two host plant species between the two groups of conditioned females. A positive conditioning event consisted of a single oviposition (egg-laying) experience into a first-instar *P. brassicae* caterpillar that was placed onto a damaged leaf of a host feeding-damaged *B. vulgaris* or *S. arvensis* plant. A negative conditioning event consisted of a 5-min host-searching experience on an induced host plant in the absence of host caterpillars or host by-products. For the positive conditioning event, each unconditioned female wasp was individually placed in a glass tube, which was then brought in close proximity to a caterpillar on a damaged leaf. The wasp was then released onto the leaf, ensuring direct contact of its antennae with a caterpillar and its products. This stimulation induced an immediate oviposition response lasting approx. 10 s. After the oviposition experience was completed, the parasitized caterpillar was removed. Between the positive and the negative experience, wasps were individually kept in glass vials for a period of 5 min. After conditioning was completed, wasps were transferred to a Petri dish (15 cm diameter) with honey and water until they were released into the tent 1 day later.

### Host-plant induction

Two days before each foraging trial, every host plant was infested with 10 first-instar *P. brassicae* caterpillars that were enclosed within a 5.5-cm diameter clip cage placed on the youngest fully grown leaf. These clip cages were removed just prior to the release of the wasps in the foraging trials. As early-instar *P. brassicae* caterpillars feed gregariously, they remained clustered on the leaf onto which they were introduced. The same procedure for *P. brassicae* infestation was followed for host plants used for the positive conditioning procedure. For the negative conditioning procedure, host plants were induced by pinching 16 small holes with a needle in the youngest fully grown leaf, followed by the application of 25 μL of *P. brassicae* regurgitant. This was done to ensure that no caterpillar-derived cues would remain on the plants that may have elicited a rewarding response in the wasps. This procedure was performed 2 days and then repeated 1 day before the conditioning procedure.

### Data analysis

#### Foraging efficiency

To assess differences in *C. glomerata* foraging efficiency, we analyzed the main and interaction effects of “habitat type” (no background vegetation/natural background vegetation) and “*C. glomerata* experience” (naïve/positive oviposition experience on *B. vulgaris*/positive oviposition experience on *S. arvensis*) on the proportion of parasitized host patches using a generalized linear model (GLM) with binomial distribution and logit-link function. The full model contained all treatment interactions, as well as experimental repetition, as fixed factors. The minimum adequate model (MAM) was determined by step-wise elimination of the highest-order least-significant term. Normality and homogeneity of variance were checked by visual inspection of the residuals.

#### Host-patch exploitation

We used the same approach to test for the degree of host patch exploitation, and analyzed the main and interaction effects of the treatments “habitat type”, “*C. glomerata* experience”, and “host-plant species” on the proportion of parasitized caterpillars per parasitized host patch (which was first averaged per tent over each plant species). It should be noted that 14 out of 108 data points consisted of so-called “structural zeroes”, because no parasitized hosts were found in these treatment combinations. These structural zeroes were omitted from the analysis.

#### Effect of learning on the orientation towards host-plant HIPVs

As the degree of host-patch exploitation was not influenced by any main or interaction effects of “habitat type” and “*C. glomerata* experience” (see the “Results” section for more details), we assessed the effect of learning on the orientation towards host-plant HIPVs by comparing the distribution of parasitized host patches over the two plant species between the different treatment groups. For the naïve *C. glomerata* females, we tested the effect of habitat type on the proportion of parasitized host patches found on *B. vulgaris* plants. For the experienced *C. glomerata* females, we tested the main and interaction effects of “habitat type” and “host plant species used for positive conditioning” on the proportion of parasitized host patches found on *B. vulgaris* plants. Data analysis followed the same approach as described above. All statistical tests were carried out in R (version 3.0.2). Post-hoc tests of interaction effects were performed with the package Phia in R, using “Holm” as the *P*-value adjustment method.

## Results

### Foraging efficiency

**Fig. 1:**
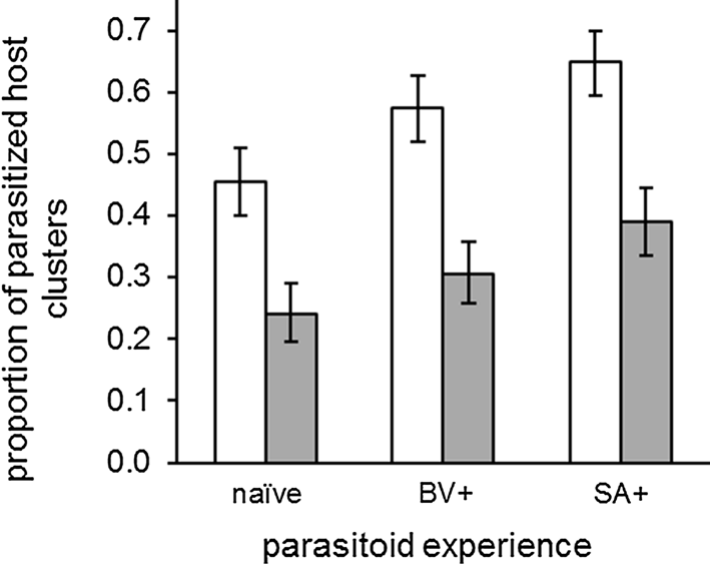
Foraging efficiency: proportion of host patches that were parasitized, averaged over each combination of the “habitat complexity” and “conditioning” treatments. Bars indicate treatment mean ± SE. *White bars* represent the habitat without background vegetation and *grey bars* represent the habitat with natural background vegetation.* naïve* = *C. glomerata* females without host-plant odour experience,* BV+* = *C. glomerata* females that received a positive conditioning event on a *B. vulgaris* plant followed by a negative conditioning event on a *S. arvensis* plant,* SA+* = *C. glomerata* females that received a positive conditioning event on a *S. arvensis* plant followed by a negative conditioning event on a *B. vulgaris* plant

The foraging efficiency of *C. glomerata* was reduced when host plants with caterpillars were surrounded by natural vegetation (Fig. 1). Within the 3-h foraging period, approximately twice as many host patches were parasitized in the absence of natural vegetation ($\chi_{1,47}^{2} = 40. 10$, *P* < 0.001) for all three “wasp experience” treatments (interaction between habitat complexity and wasp experience was not significant: $\chi_{ 2, 4 7}^{2} = 0. 1 4 6$, *P* = 0.929). Overall, 28 % fewer host patches were parasitized by naïve *C. glomerata* females than by females that received an oviposition experience into a single *P. brassicae* caterpillar on a host-damaged plant 1 day prior to the foraging trial ($\chi_{ 2, 4 7}^{2} = 1 3. 4 1$, *P* = 0.001).

### Host-patch exploitation

**Fig. 2:**
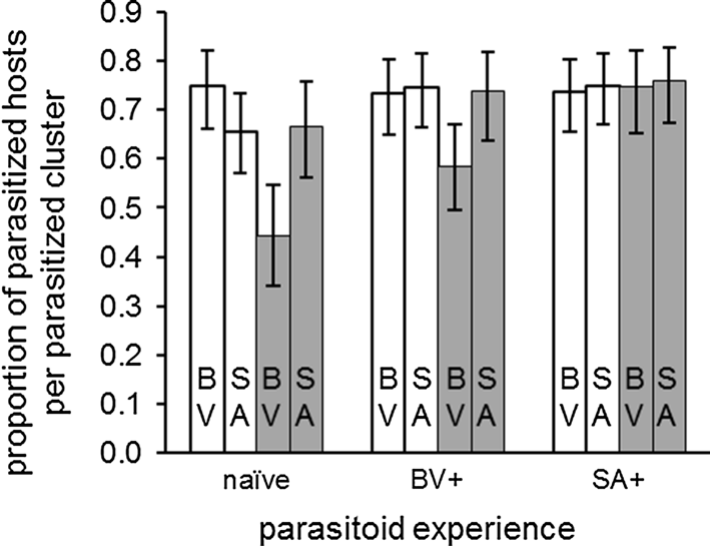
Host-patch exploitation: proportion of parasitized caterpillars per parasitized host patch.* Bars* indicate treatment mean ± SE. *White bars* represent the habitat without background vegetation and *grey bars* represent the habitat with natural background vegetation.* SA and BV within bars* represent *S. arvensis* plants and *B. vulgaris* plants, respectively.* naïve* = *C. glomerata* females without host plant odour experience,* BV+* = *C. glomerata* females that received a positive conditioning event on a *B. vulgaris* plant followed by a negative conditioning event on a *S. arvensis* plant,* SA+* = *C. glomerata* females that received a positive conditioning event on a *S. arvensis* plant followed by a negative conditioning event on a *B. vulgaris* plant

In contrast, the number of parasitized hosts per parasitized host patch did not vary with habitat type, *C. glomerata* experience or host-plant species (Fig. 2; $\chi_{ 1, 9 2}^{2} = 1. 9 6$, *P* = 0.162; $\chi_{ 2, 8 7}^{2} = 3. 6 2$, *P* = 0.164 and $\chi_{ 1, 9 1}^{2} = 0. 8 2 6$, *P* = 0.364, respectively), and there were no significant interactions between these treatment factors.

### Effect of learning on the orientation towards host-plant HIPVs

**Fig. 3:**
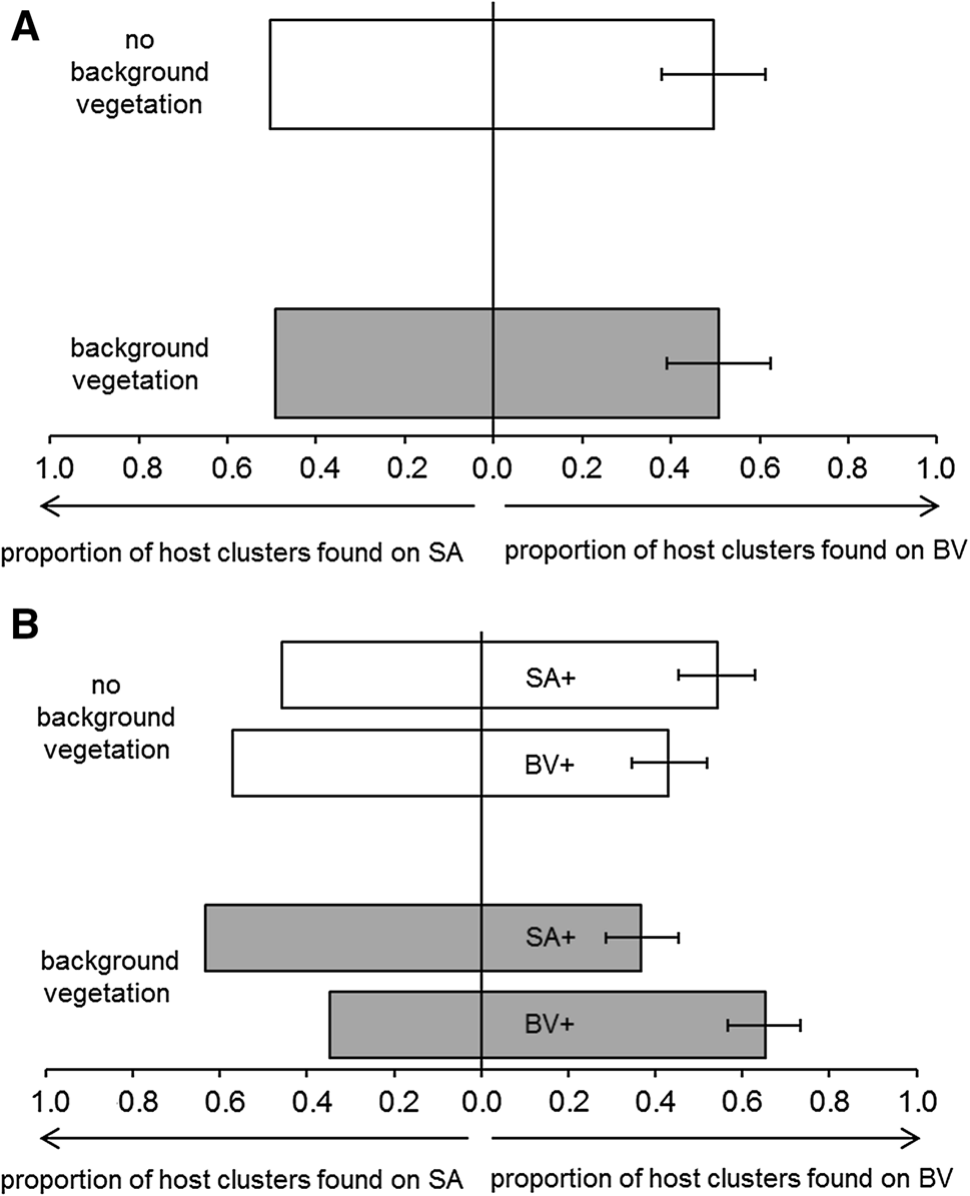
Effect of learning on the orientation towards host-plant HIPVs: distribution of parasitized host patches over *B. vulgaris* and *S. arvensis* plants for the group of naïve females (**a**) and the two groups of experienced females (**b**).* Bars* indicate treatment mean ± SE. *White bars* represent the habitat without background vegetation and *grey bars* represent the habitat with natural background vegetation.* BV+* = *C. glomerata* females that received a positive conditioning event on a *B. vulgaris* plant followed by a negative conditioning event on a *S. arvensis* plant,* SA+* = *C. glomerata* females that received a positive conditioning event on a *S. arvensis* plant followed by a negative conditioning event on a *B. vulgaris* plant

For naïve *C. glomerata* females, the presence of background vegetation did not affect the distribution of parasitized host patches over the two host-plant species (Fig. 3a; $\chi_{1,16}^{2} = 0.00 4$, *P* = 0.947). Moreover, in neither of the two habitat types did the percentage of parasitized host patches found on *B. vulgaris* deviate from 50 % (two-tailed binomial test: no vegetation, *P* = 0.568; background vegetation, *P* = 1.000). With respect to experienced *C. glomerata* females, we found a significant interaction between “habitat type” and “host plant species used for positive conditioning” (Fig. 3b; $\chi_{ 1, 3 2}^{2} = 5. 1 9$, *P* = 0.023). Only in the presence of natural background vegetation was a larger proportion of parasitized host patches found on the host-plant species used for positive conditioning ($\chi_{1,37}^{2} = 5. 1 3$, *P* = 0.047). In the habitat without background vegetation, the proportion of parasitized host patches did not differ between the two host-plant species ($\chi_{ 1, 3 2 }^{2} = 0. 8 1$, *P* = 0.369).

## Discussion

Our results show that natural vegetation surrounding host-infested plants reduces the host-encounter rate of *C. glomerata* females. This may be attributed to chemical and/or structural masking of HIPVs (Casas and Djemai 2002; Gols et al. 2005; Obermaier et al. 2008; Randlkofer et al. 2010; Wäschke et al. 2013). Moreover, we found that associative learning of host-plant HIPVs caused an overall increase in the proportion of host patches that were parasitized. This finding presents an important addition to the current literature, as studies showing the benefits of associative learning on foraging efficiency in (semi-) field situations are still scarce (but see Papaj and Vet 1990; Raine and Chittka 2008; Zrelec et al. 2013). For example, it has been demonstrated that olfactory learning by the parasitoid *Leptopilina heterotoma* increased both the chance of finding *Drosophila* hosts under field conditions and the speed with which they were found (Papaj and Vet 1990). Also, a study comparing different colonies of bumble bees reported a positive correlation between learning speed and natural foraging success (Raine and Chittka 2008).

Although previous studies have indicated that any negative impacts of habitat complexity on parasitoid foraging efficiency disappear after the acquisition of oviposition experience in the presence of host-plant HIPVs (Bukovinszky et al. 2007; Perfecto and Vet 2003), we did not find such an interaction effect between learning and habitat complexity. Regardless of wasp experience, the proportion of parasitized host patches was reduced by half in the presence of natural vegetation. The absence of this interaction effect may be explained as follows. First, in spite of its statistical significance, the impact of learning on foraging efficiency was relatively weak, making it harder to detect any interactions between learning and habitat complexity. This may have been because parasitoid foraging took place in tents containing a mixed configuration of two equally rewarding host-infested plant species, while wasp conditioning was geared towards the association of only one of the plant species with a reward. As a consequence, learning to focus on only half of the plants present in each tent may have put experienced wasps at a relative disadvantage compared to naïve wasps, which were attracted to all plants. Second, we conditioned females on isolated host-infested plants in the absence of the background odour from vegetation. Although still unstudied, it may be that there is a benefit of learning host-plant HIPVs in the right context, i.e. in the type of vegetation in which the parasitoid will also subsequently forage for hosts, especially for parasitoids that forage in more chemically complex environments.

Interestingly, a relative increase in the response to the odour of the host-plant species used for positive conditioning was only detected when host-infested plants grew in the presence of natural vegetation. For naïve *Cotesiaglomerata* females, no difference in orientation towards the two host-plant species was detected in either of the habitat types; in both the presence and absence of background vegetation, the parasitized host patches were equally distributed over *S. arvensis* and *B. vulgaris* plants. As far as we are aware, this is the first time that an increase in orientation specifically towards the learned odour has been demonstrated under more natural circumstances, i.e. with host-infested plants growing within a background of natural vegetation. This clearly shows that a chemically complex environment does not impede experienced parasitoids from orienting towards host-plant HIPVs. Recent work suggests that parasitoids can achieve high odour discrimination abilities in complex field situations through movement (Meiners et al., unpublished results in Wäschke et al. 2013). They showed that the presence of non-host-plant odours completely impedes the recognition of the host-plant odour by the egg parasitoid *Oomyzus galerucivorus* when both odours are presented simultaneously from the same angle, but not when the two odours are separated by 1 cm.

At first sight, it seems curious that no effect of learning on the distribution of parasitized host patches between the two host-plant species was detected in the habitat without background vegetation. Yet, at the point of 50 % host-patch parasitization, it may be that all of the plants of one host-plant species had been visited by parasitoids. Any further parasitization would then have lowered the chance of detecting a difference in the degree of parasitization between the two host-plant species. Keeping the foraging time between the two habitats equal to permit a comparison of foraging efficiency resulted in a relatively high proportion of parasitized host patches in the habitat without background vegetation (with 70 % of the tents containing 50 % or more parasitized host patches, against 19 % of the tents in the habitat with natural background vegetation). To better compare the orientation of parasitoids towards the HIPVs of host-plant species growing in diverse habitats, it would be ideal to monitor the foraging decisions of individual wasps, preferably at larger spatial scales where hosts are more sparsely distributed. However, this has never been done under natural circumstances and may be especially challenging in habitats with dense vegetation. Alternatively, the percentage of plants containing parasitized host patches could be kept similar between habitats, and below 50 %, by reducing parasitoid foraging time in the more simple habitats and/or by challenging the parasitoids more by reducing the ratio of host-infested to intact host plants.

Parasitoids follow a step-wise hierarchical process to locate suitable hosts (Vinson 1976). While the first challenge that a parasitoid female faces is finding a suitable habitat following eclosion (Fei et al. 2014), our experiments focused on the influence of natural vegetation on parasitoid host location within a habitat. In this context, it is important to note that plant species diversity may have an influence on both of these aspects, and may even do so in contrasting ways (Schroeder and Hilker 2008). In some cases, a background odour may repel insects, causing them to stay away from the vegetation patch, and may mask host-plant odours (Hori and Komatsu 1997; Mauchline et al. 2005). Sometimes, however, less attractive plants may enhance parasitoid foraging efficiency for host-infested plants within a patch (Soler et al. 2007). In other cases, plants surrounding host-infested plants may attract parasitoids, thus stimulating parasitoids to enter the vegetation patch while at the same time reducing parasitoid foraging efficiency within the patch (Gols et al. 2005; Perfecto and Vet 2003). In other cases, a background odour is neither repellent nor attractive but may still mask host-plant HIPVs. This may then result in reduced attraction to the vegetation patch as well as reduced attraction to host-infested plants (Monteith 1960; Visser and Ave 1978). It is therefore important to investigate parasitoid foraging behaviour in response to vegetation diversity at these different scales, and, in addition to the current study, future studies should shed more light on the factors that determine habitat selection in *Cotesia glomerata*.

In conclusion, we found that the presence of natural vegetation surrounding host-infested plants reduces the foraging efficiency of *C. glomerata*, while it did not prevent experienced *C. glomerata* females from parasitizing more host patches on the host plant species for which they had previously learnt the HIPVs. This indicates that, in more complex natural habitats too, *C. glomerata* females can optimize their foraging behaviour by orienting specifically to host-plant HIPVs in the presence of which they had previously had a positive experience. Our results emphasize that it is essential to not only consider the host and its food plant, but also the influence of the surrounding vegetation when studying different aspects of parasitoid foraging behaviour.

### Author contribution statement

HMK, TSH and ALR conceived and designed the experiments. HMK, ALR, PM and DM performed the experiments. HMK analysed the data and wrote the manuscript. JAH, TSH, LEMV and RG provided advice on the experimental design and the manuscript.
